# *Tmem26* Is Dynamically Expressed during Palate and Limb Development but Is Not Required for Embryonic Survival

**DOI:** 10.1371/journal.pone.0025228

**Published:** 2011-09-29

**Authors:** Liam Town, Edwina McGlinn, Tara-Lynne Davidson, Catherine M. Browne, Kallayanee Chawengsaksophak, Peter Koopman, Joy M. Richman, Carol Wicking

**Affiliations:** 1 Institute for Molecular Bioscience, The University of Queensland, Brisbane, Australia; 2 Life Sciences Institute, Department of Oral Health Sciences, University of British Columbia, Vancouver, Canada; CNRS, France

## Abstract

The *Tmem26* gene encodes a novel protein that we have previously shown to be regulated by hedgehog signalling in the mouse limb. We now report that *Tmem26* expression is spatially and temporally restricted in other regions of the mouse embryo, most notably the facial primordia. In particular, *Tmem26* expression in the mesenchyme of the maxillary and nasal prominences is coincident with fusion of the primary palate. In the secondary palate, *Tmem26* is expressed in the palatal shelves during their growth and fusion but is downregulated once fusion is complete. Expression was also detected at the midline of the expanding mandible and at the tips of the eyelids as they migrate across the cornea. Given the spatio-temporally restricted expression of *Tmem26*, we sought to uncover a functional role in embryonic development through targeted gene inactivation in the mouse. However, ubiquitous inactivation of *Tmem26* led to no overt phenotype in the resulting embryos or adult mice, suggesting that TMEM26 function is dispensable for embryonic survival.

## Introduction

*Transmembrane protein 26 (Tmem26)* encodes a 41.6 kD transmembrane protein that is conserved throughout much of animal evolution but whose function remains unknown. We initially demonstrated that *Tmem26* expression is regulated by the GLI3 transcription factor in the murine anterior limb at 11.5 dpc [1]. GLI3 is primarily responsible for repressing hedgehog signalling, and the pivotal role of sonic hedgehog in determining digit number and identity is mediated through regulation of the cleavage of the full-length GLI3 isoform to a truncated transcriptional repressor [2], [3], [4]. *Tmem26* expression is convincingly downregulated in response to loss of GLI3 repressor activity on the anterior limb bud [1], suggesting that it may also form part of the complex molecular circuitry governing limb patterning. However, very little is known about *Tmem26* expression in other tissues or at different embryonic stages.

Here we demonstrate that expression of *Tmem26* is temporally and spatially restricted at a range of mid-gestational embryonic stages in mice. Most notably, we found highly restricted expression in the developing facial prominences and palatal structures in a manner suggesting involvement in formation of both the primary and secondary palates. Despite this, ubiquitous inactivation of *Tmem26* in the mouse led to no overt developmental or adult anomalies, suggesting that TMEM26 is not required for embryonic development.

## Results and Discussion

### Tmem26 structure and evolution

**Figure 1:**
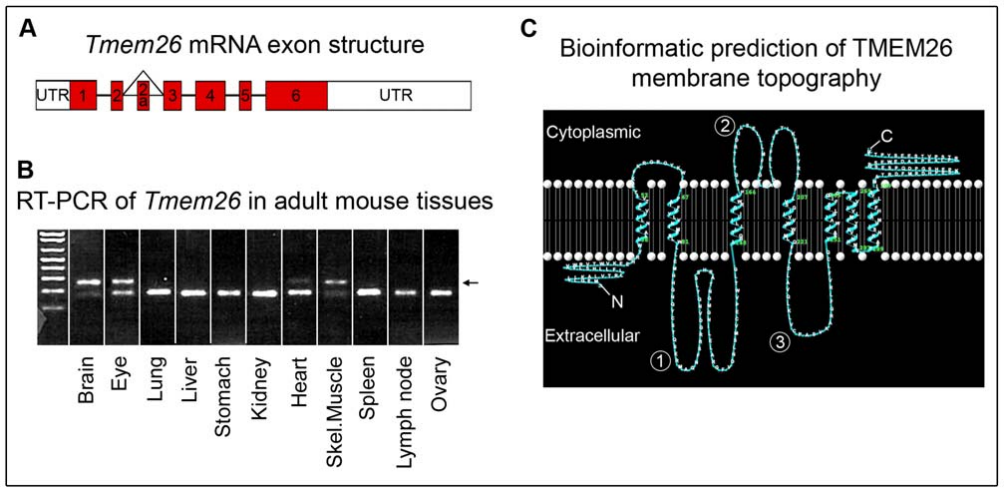
Predicted gene and protein structure. (**A**) Predicted exon structure of *Tmem26*. (**B**) RT-PCR across exon2, showing the main *Tmem26* transcript incorporating exon2 only, and a minor variant generated by alternative splicing in some tissues (arrow), which incorporates an extra exon (2a). (**C**) The topographic prediction for TMEM26 generated by the program SVTtm. Other predictions vary dependent on the presence or absence of a leader sequence and the number of transmembrane domains. 1,2,3 – non-membrane loops; C-terminus, N-terminus.

*Tmem26* localises to chromosome 10 in both mice and humans. In mice, the transcript most commonly represented in expressed sequence tags (EST) databases is approximately 6 kb and formed from six exons (Fig. 1A). In addition to this primary transcript, there is likely to be an additional splice variant that incorporates an 82 bp exon (exon 2a) after exon 2 in mice (Fig. 1A). Insertion of exon 2a causes a frameshift and introduces an early stop codon in exon 3. However, this variant is represented by only a few sequences in mouse EST databases. Reverse transcriptase-polymerase chain reaction (RT-PCR) of 13.5 dpc whole mouse embryo RNA failed to detect the splice variant, although analysis of various adult tissues detected both the primary and the variant mRNAs in whole brain, eye, skeletal muscle and heart (Fig. 1B). In humans, four splice variants are represented in Ensembl databases (www.ensembl.org - ENSG00000196932). The primary human transcript, which corresponds to the primary mouse transcript, is highly represented in EST and mRNA databases, suggesting that it is the major transcript expressed. All other human splice variants encode proteins with large truncations, frameshifts or early stop codons. No known human splice variants reflect the exon 2a mouse splice variant.

*Tmem26* encodes a predicted protein consisting of 367 and 368 residues in mice and humans respectively, with an estimated mass of 41.6 kilodaltons (kDa). Online bioinformatic databases do not predict any recognisable protein structural domains within TMEM26, although several hydrophobic regions thought to act as transmembrane domains are predicted. The *Tmem26* gene is also found in *Drosophila*, and in most animal species studied appears to be represented by a single orthologue; exceptions include zebrafish (*Danio rerio*) and sea squirts (*Ciona intestinalis* and *Ciona savignyi*) which have multiple paralogues.

Structural predictions using online transmembrane prediction programs (SVMtm [5], TmPred, TMHMM [6], HMMTOP [7] and Swissprot) suggest that TMEM26 contains between 5 and 8 transmembrane domains, with the most commonly predicted number being 7. The variation between predictions often depends on the presence or absence of a leader sequence (which replaces one transmembrane domain) and the number of domains within the C-terminal region. The topographic prediction generated by SVMtm, which recognises leader sequences of known proteins more accurately than alternative membrane topography prediction programs [5], is shown in Fig. 1C.

### Embryonic expression

**Figure 2:**
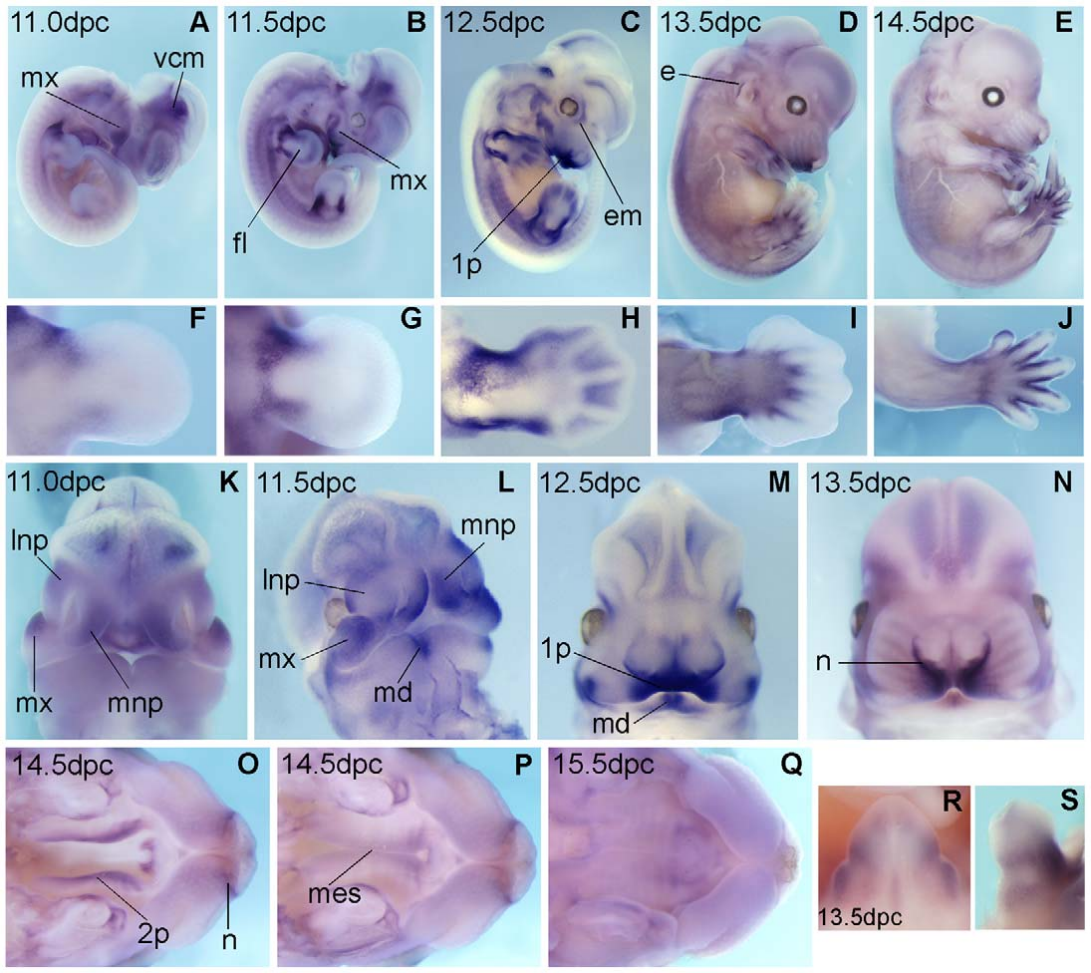
Expression of *Tmem26* in wild-type embryos by whole mount in situ hybridisation analysis. (**A–E**) Whole embryos aged 11.0 dpc-14.5 dpc. No expression was detected before 11.0 dpc. (**F–J**) Forelimbs of corresponding embryos shown in **A–E**, dorsal view, limb anterior is to the top. (**K,L**) At 11.0 and 11.5 dpc *Tmem26* expression in the facial prominences is restricted primarily to mesenchyme underlying areas of facial prominence fusion and merging. (**M**) At 12.5 dpc striking expression is observed in the primary palate, and expression remains at the midline of the mandible. (**N**) After fusion is complete at 13.5 dpc, expression is restricted to maxillary tissue at the distal tip of the snout. (**O,P**) The developing secondary palate at 14.5 dpc, taken from below with the mandible removed, one showing the palatal shelves wide apart, and one as the shelves are coming together to fuse. *Tmem26* is expressed in the palatal shelves prior to fusion but downregulated upon palate fusion, most obvious at 15.5 dpc (**Q**). (**R**) Ventral and (**S**) lateral views of a 13.5 dpc genital tubercle. Dorsal-ventral and anterior-posterior axes are indicated. 1p-primary palate, 2p-secondary palatal shelves, e-external ear, em-extraoccular musculature, fl-forelimb, lnp-lateral nasal prominence, mes-medial epithelial seam, md-mandible, mnp-medial nasal prominence, mx-maxillary prominence, n-nasal opening, vcm-ventral cephalic mesenchyme.

The expression profile of *Tmem26* mRNA in the murine embryo was investigated at a range of mid-gestational stages using whole mount in situ hybridisation (WISH). At embryonic stages 8.5, 9.5 and 10.5 dpc, *Tmem26* mRNA was not detected by WISH at levels above background (data not shown). At 11.0 dpc, *Tmem26* expression was detected in the mesenchyme of the anterior and posterior proximal limb bud, at the autopod/zeugopod boundary (Fig. 2A,F). By 11.5 dpc, forelimb and hindlimb displayed increased expression within these domains (Fig. 2B,G). We have shown previously that the anterior domain is lost in *Gli3^Xt/Xt^* mice that are considered null for *Gli3* (see probe BF147423 in [1]). At 12.5 dpc, *Tmem26* expression was detected throughout much of the interdigital space mesenchyme but by 13.5 dpc, this was limited to the mesenchyme at the margins of the digits (Fig. 2H–J).

**Figure 3:**
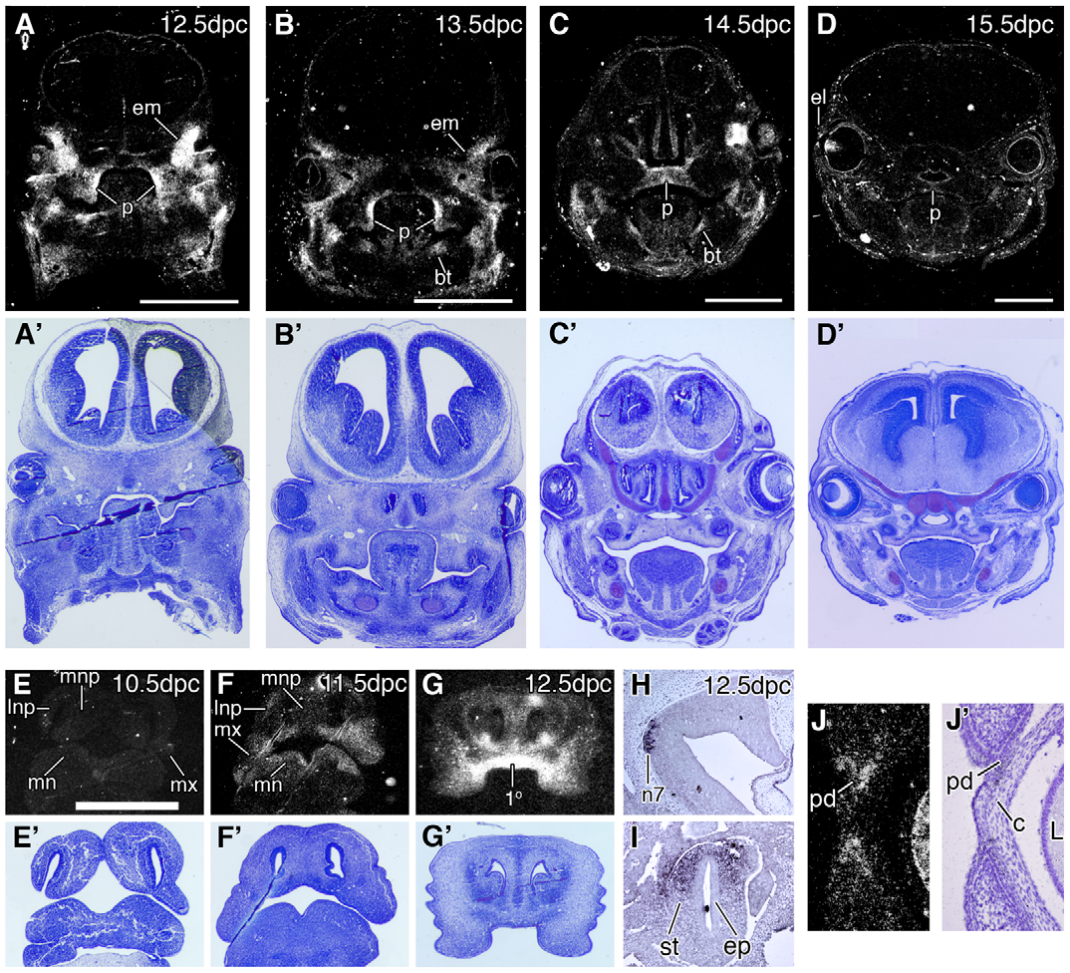
Expression of *Tmem26* in wild-type embryos by section in situ hybridisation. (**A–D, E–G,J**) radioisotopic; (**H,I**) DIG detection. (**A–D**) Transverse sections through the secondary palate at indicated stages. At 12.5 dpc and 13.5 dpc *Tmem26* is expressed in mesenchyme of the vertical palatal shelves. At 14.5 dpc the shelves have fused but the medial epithelial seam still remains and expression within the mesenchyme is apparent. By 15.5 dpc the epithelial seam is absent and *Tmem26* expression is virtually undetectable. (**A′–D′**) Corresponding serial sections stained with toluidine blue. (**E–G**) Sections through the facial prominences and primary palate region at indicated stages. *Tmem26* is not detected at 10.5 dpc but is upregulated at 11.5 dpc and 12.5 dpc. (**E′–G′**) Corresponding sections stained with toluidine blue. (**H**) Parasagittal section through the lateral hindbrain at 12.5 dpc, showing expression in the nucleus of the seventh facial nerve region within the pons. (**I**) Longitudinal section through the stomach at 11.5 dpc, showing expression in the anterior stomach mesenchyme. (**J**) Magnification of the left eyelid from **D**, (**J′**) same section counterstained with haematoxylin. 1p-primary palate, bt-mesencyme at the base of the tongue, c-cornea, el-eyelid, em-extraocular mesenchyme and developing extrinsic muscles of the eye, ep-epithelim, L-eye lens, lnp-lateral nasal prominence, n7-nucleus of the seventh facial nerve, mn-mandible, mnp-medial nasal prominence, mx-maxillary prominence, ns-nasal septum, p-palatal shelf, pd-eyelid periderm, st-stomach mesenchyme. Size bars indicate 1 mm.

By 11.5 dpc, we also detected expression of *Tmem26* in a broad domain across the surface of the flank, and in craniofacial structures as discussed in more detail below. Other sites of expression revealed by either WISH or section in situ hybridisation include the anterior mesenchyme of the stomach at 11.5 dpc (Fig. 3I), the preputial swellings and proximo-lateral glans of the genital tubercle from 11.5 dpc to 15.5 dpc (Fig. 2R,S and data not shown), and a defined region of the hindbrain from 12.5 dpc (Fig. 3H). This brain region likely corresponds to the nucleus of the seventh cranial nerve (the facial nerve) in the pons.

### Embryonic craniofacial expression

The mammalian face is mostly derived from the frontonasal bulge and pharyngeal arches. A critical process in development of the face is the morphogenesis of these progenitor structures into the lateral nasal, medial nasal, maxillary and mandibular prominences, and the subsequent expansion and fusion of the prominences to form the primary and secondary palates and the lower jaw. Understanding these processes has considerable importance for human health, as failure of the facial prominences to fuse is often the underlying cause of midfacial clefting defects including cleft lip with or without cleft palate - the most common form of human craniofacial birth defect [8], [9], [10].

At 11.0 dpc *Tmem26* expression was first evident diffusely throughout the medial and lateral nasal prominences (Fig. 2K). At 11.5 dpc, the posterior region of the medial nasal prominence begins to fuse with the lateral nasal prominence and the maxillary prominence to form the upper lip and primary palate. *Tmem26* was found to be expressed in each of these three prominences at this time point in the underlying mesenchyme adjacent to the regions of fusion (Fig. 2L). It was also expressed along the posterior margin of the maxillary prominence and along the medial surface of each medial nasal prominence near the region where they will ultimately merge at the midline (Fig. 2L). At 12.5 dpc, *Tmem26* became strikingly restricted within the primary palate (Fig. 2M). At 13.5 dpc, when primary palate formation is complete, *Tmem26* expression was restricted to the distal tips of the maxillae and the maxillary margins of the nostrils (Fig. 2N). A zone of expression was also present along the oral edge of the mandibular prominence, from 11.5 dpc to 13.5 dpc (Fig. 2K–M). This dynamic expression in the facial prominences and primary palate was confirmed by radioisotopic in situ hybridisation of transverse sections through the embryonic head (Fig. 3E–G′). In particular, no expression was detected prior to 11.0 dpc. Onset of *Tmem26* expression at this late stage suggests that *Tmem26* may not be involved in jaw patterning but could play a role in lip or secondary palate fusion.

The striking expression of *Tmem26* in the primary palate prompted us to investigate the secondary palate, the formation of which is distinct from that of the primary palate but shares many common genetic pathways and cellular mechanisms. The palatal shelves emerge from the oral surface of the maxillae at 12.5 dpc. The shelves expand, reorient and ultimately fuse around 14.5 dpc to divide the oral from the nasal cavity. By WISH we detected *Tmem26* expression in the palatal shelves prior to fusion (Fig. 2O). However, as regions of the palate begin to fuse at 14.5 dpc, *Tmem26* appeared to be downregulated in the neighbouring mesenchyme (Fig. 2P). By 15.5 dpc, when fusion is essentially complete, expression in the secondary palate is greatly reduced (Fig. 2Q). This expression was also confirmed by radioisotopic section in situ hybridisation (Fig. 3A–D′). The mesenchyme of the vertical palatal shelves expressed *Tmem26* from the time of emergence at 12.5 dpc, through 13.5 dpc (Fig. 3A,B). By 14.5 dpc when the palatal shelves have reoriented in most embryos, expression was still evident throughout the mesenchyme (Fig. 3C), but this was rapidly downregulated following fusion and degeneration of the medial epithelial seam at 15.5 dpc (Fig. 3D).

Other sites of *Tmem26* expression within the craniofacial complex included the nasal conchae (not shown) and septum, extra-ocular mesenchyme, extrinsic muscles of the eye, and the base of the tongue (Fig. 3A–D′). At 15.5 dpc, *Tmem26* expression was also detected in the ectodermally-derived periderm and epithelia at the tip of the developing eyelid (Fig. 3J). At this time point, the eyelids are migrating across the cornea to fuse at the equator. Expression was not detected in the developing teeth or Meckel's cartilage.

### Adult expression

**Figure 4:**
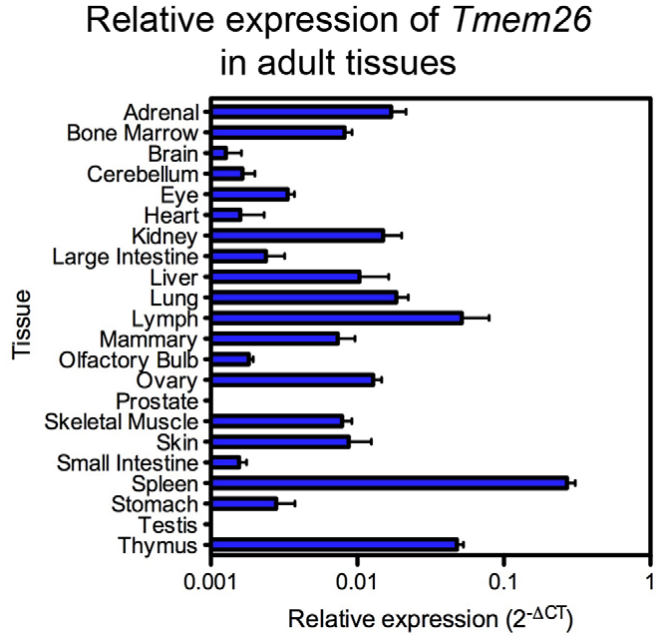
Expression of *Tmem26* as determined by qRT-PCR. Graph shows the mean 2^−ΔCT^ and the standard error of the mean (SEM).

Publicly available array data (http://symatlas.gnf.org/SymAtlas/symform) demonstrates low level expression of *Tmem26* in a wide range of human and mouse adult tissues. Widespread expression was confirmed by real-time quantitative RT-PCR (qRT-PCR) in a range of adult mouse tissues, although expression was not detected in prostate or testis, and enhanced levels were detected in mesenteric lymph nodes, spleen and thymus (Fig. 4). This profile of expression suggests a possible role for TMEM26 in immune function.

### Generation of floxed Tmem26 mice

**Figure 5:**
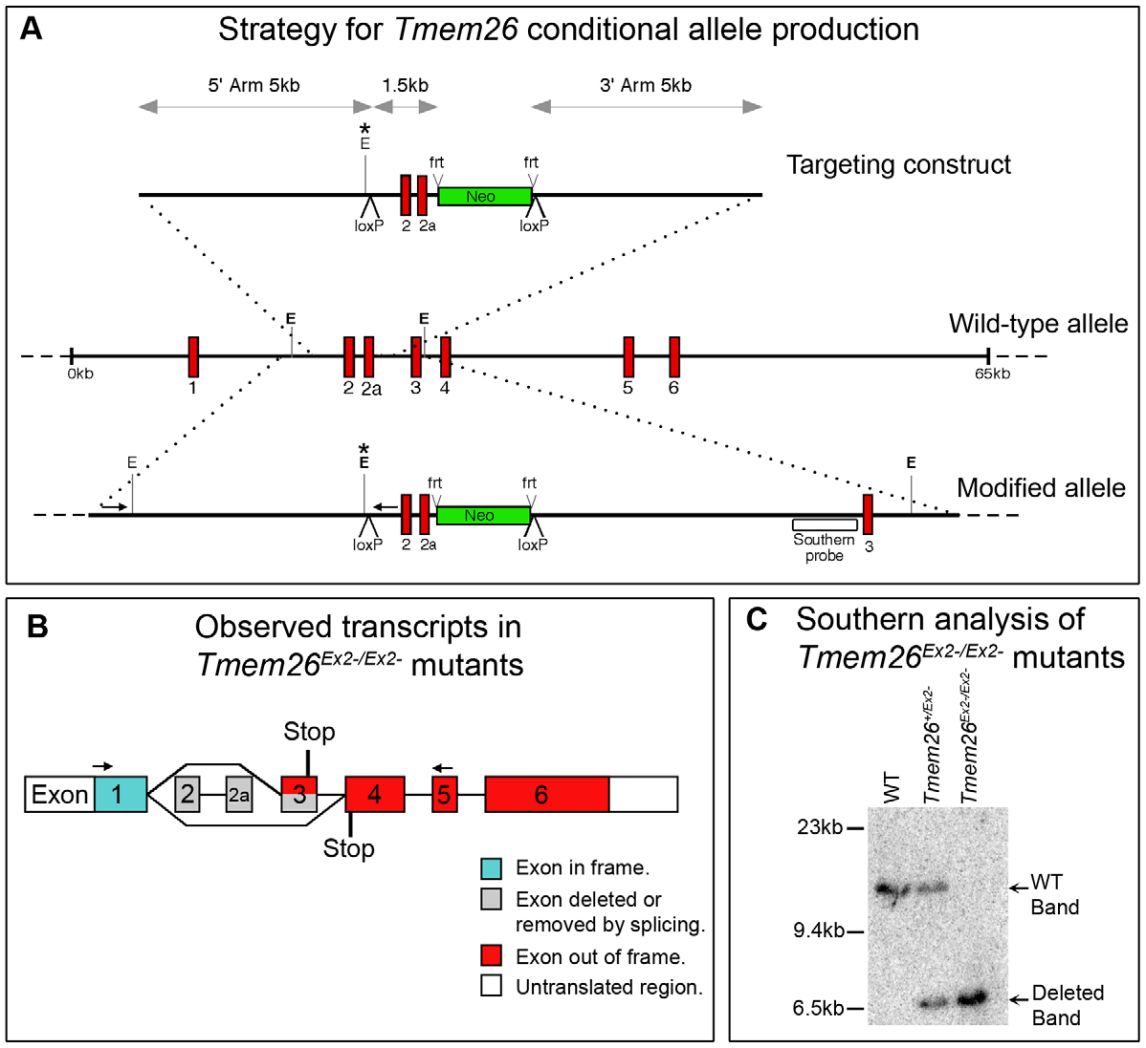
Gene targeting strategy for the *Tmem26* locus. (**A**) Targeting construct for *Tmem26* conditional inactivation compared to the wild type allele and the floxed *Tmem26* allele after homologous recombination. In the presence of Cre recombinase, the region between *LoxP* sites will be excised, including exon 2, exon 2a (red) and the neomycin selection cassette (green). The neomycin selection cassette can be independently excised by *Flp* recombinase (*frt* sites). A Southern probe (open box) and PCR primers (small arrows) external to the targeting construct were used to screen stem cells for successful homologous recombination. An *Eco*RV site (E*) was introduced with the targeting construct and was diagnostic during Southern and PCR assays for 5′ *LoxP* integration. (**B**) Two transcripts were detected, one lacking exons 2 and 2a as predicted, and another variant in which exon 3 was also absent. In both cases exon 1 is retained, and the exons after the deleted exons are out of frame. A premature stop codon is introduced in both cases (**C**) Southern blot showing the wild type and mutant band arising from Cre-mediated excision (*Tmem26^Ex2−^*).

To investigate the functional role of TMEM26 in various tissues during embryonic development we generated mice that would allow conditional inactivation of *Tmem26* in a temporal and spatial manner. *LoxP* recombination sequences were inserted on either side of a genomic region of ∼1.5 kb containing exon 2 and the splice variant exon 2a (Fig. 5A). Deletion of exon 2 would remove a single predicted transmembrane domain and introduce a frameshift to the transcript, resulting in a premature stop codon in exon 3 (Fig. 5B). The targeting vector contained 5′ and 3′ recombination arms each of approximately 5 kb, as well as a neomycin selection cassette flanked by *Frt* sites for removal of this cassette if required.

The *Tmem26* targeting construct was electroporated into C2 embryonic stem cells (ES) derived from the C57BL/6 mouse strain. The C57BL/6 background was selected because of its susceptibility to craniofacial defects in a number of alternative mouse models, such as *Gli3* and *Treacle* mutants [11], [12]. Resistant colonies were screened for targeted recombination using PCR and Southern blot analysis. The 768 bp hybridisation probe binds the genomic DNA 3′ of the targeting vector homologous region (Fig. 5A). Hybridisation of this probe to *Eco*RV digested DNA identifies the wild type allele (*Tmem26^+^*), the targeted allele (*Tmem26^c^*), and also the deleted allele produced by CRE-mediated recombination (*Tmem26^Ex2−^*; Fig. 5C). Correctly targeted ES cells were injected into C57BL/6 blastocysts, and chimeric offspring identified by PCR. Further breeding established germline transmission in a number of lines.

### Ubiquitous inactivation of Tmem26

*Tmem26^c/c^* mice were crossed to CMV-*Cre* mice [13] expressing Cre recombinase ubiquitously, resulting in inactivation of *Tmem26* in all cells including germ cells. Mice homozygous for the deleted allele in subsequent generations were therefore *Tmem26* mutants (*Tmem26^Ex2−/Ex2−^*). Surprisingly, these animals were viable and fertile as adults and showed no detectable evidence of abnormal embryonic development. Unavailability of an antibody that reliably detects TMEM26 precluded confirmation of loss of protein in these mice. However, Southern analysis confirmed that the structure of the *Tmem26^Ex2−^* allele was as expected (Fig. 5C). Furthermore, sequencing of PCR products generated for genotyping of null and floxed mice revealed that the sequence surrounding both 3′ and 5′ LoxP sites was as predicted. To examine the transcriptional products of the *Tmem26^Ex2−^* allele, we used RT-PCR to amplify across the introduced mutation from mRNA derived from adult liver and brain. Two forward primers were designed within exon 1 and reverse primers were designed within exon 3, 4 and 5, thus allowing 6 separate PCR reactions that amplify regions that include exon 2. All PCR products were band purified and sequenced. Two *Tmem26* transcripts were amplified specifically from *Tmem26^Ex2−/Ex2−^* tissues. Sequencing revealed that the larger of these bands represented the expected mutant transcript lacking exon 2. The smaller band lacked both exons 2 and 3, indicating that deletion of exon 2 induced a splice variant that contains exons 1, 4, 5 and 6 (Fig. 5B). Sequencing demonstrated that both of these *Tmem26^Ex2−/Ex2−^* mRNA isoforms encode proteins in which the region encoded by exon 1 is in-frame, but is followed by nonsense sequence and a premature stop codon, again confirming correct targeting of the *Tmem26* locus.

Consistent with the viability of these mice as adults, genotypes of animals born from *Tmem26^+/Ex2−^* heterozygous matings segregated in approximately Mendelian ratios (*Tmem26^+/+^* 26 mice; *Tmem26^+/Ex2−^* 65 mice; *Tmem26^Ex2−/Ex2−^* 39 mice), indicating that the knockout allele does not cause embryonic or perinatal lethality. These data suggest that *Tmem26* is not essential for embryonic survival. The lack of embryonic phenotype is unlikely to be due to functional compensation by a related gene since our bioinformatic analysis revealed no evidence of paralogous genes in the mouse genome. It is possible that truncated proteins could be produced from the mutant transcript and may retain partial or complete *Tmem26* function. This is a consideration common to most conditional knockout models. However, if proteins were produced from *Tmem26^Ex2−/Ex2−^* transcripts, we consider it unlikely that they would retain significant function, as all possible products would be severely truncated, inhibited in their insertion and orientation within the membrane or lacking large regions of highly conserved sequence.

**Figure 6:**
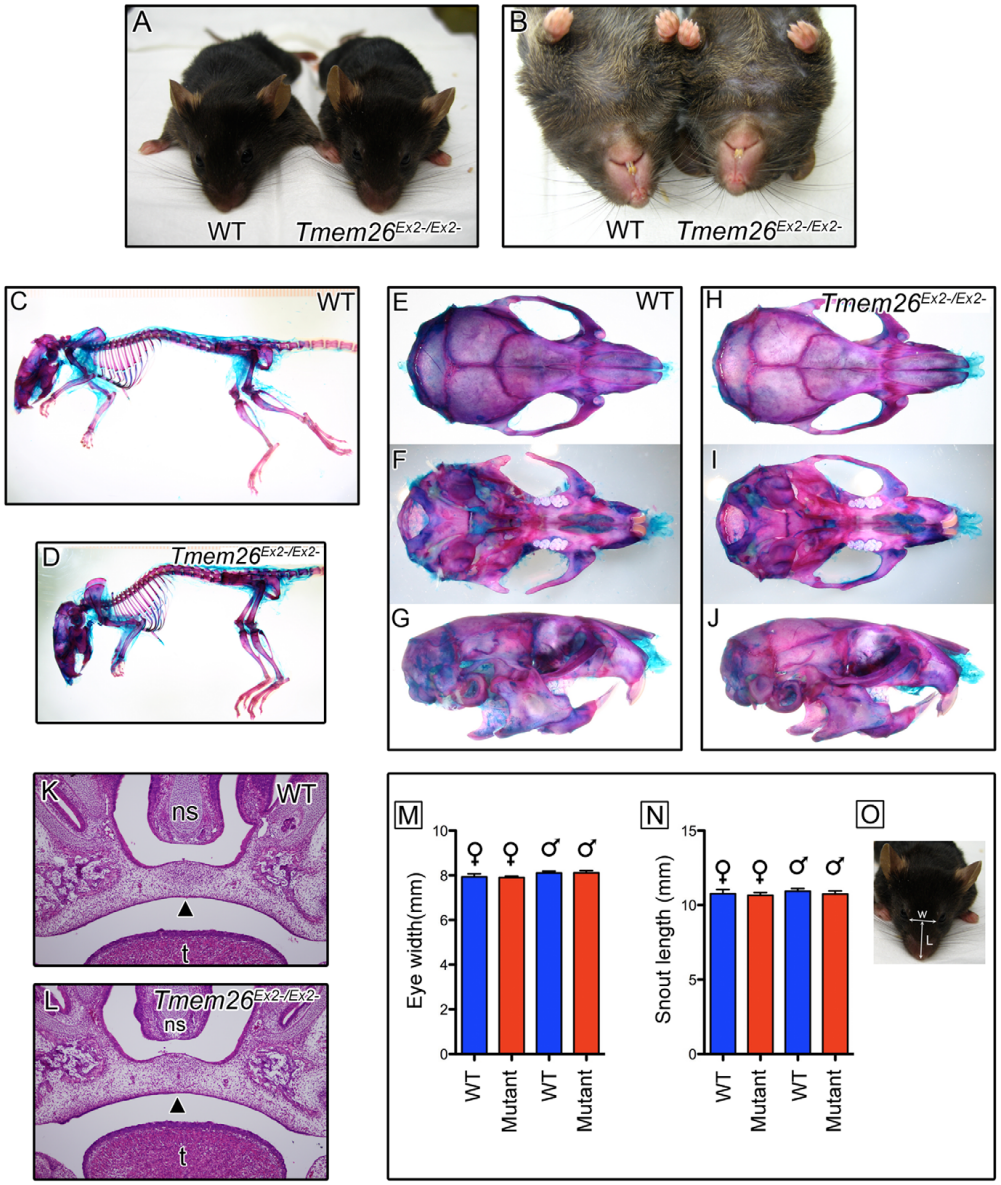
*Tmem26^Ex2−/Ex2−^* mice appear phenotypically normal. (**A**) Dorsal and (**B**) ventral views of adult WT and *Tmem26^Ex2−/Ex2−^* littermate mice. (**C–J**) Alcian blue/alizarin red staining of WT and *Tmem26^Ex2−/Ex2−^* adult littermate skeletal preparations. (**C**) and (**D**) are at the same magnification. (**E,H**) Dorsal, (**F,I**) ventral and (**G,J**) lateral views of skull. In (**F,I**) the mandible has been removed to allow visualisation of the palate. (**K,L**) Transverse sections through the secondary palate of 15.5 dpc WT and *Tmem26^Ex2−/Ex2−^* embryos. (**M**) Mean distance between the inner canthi of the eyes, and (**N**) mean length of the snout were compared between WT and *Tmem26^Ex2−/Ex2−^* adult mice (n = 18 female mice, 20 male mice per genotype). (**O**) diagram showing the measurements taken. L-snout length; w-distance between the eyes. Error bars represent standard error of the mean (SEM), statistical analysis (Student's t-test) revealed no significant difference.

Comparison of mean weight of *Tmem26^Ex2−/Ex2^* and WT littermates above 3 months of age revealed no significant differences (data not shown). The expression pattern of *Tmem26* predicts possible defects in the limbs and craniofacial complex. However, skeletal preparations of adult *Tmem26^Ex2−/Ex2−^* and WT mice revealed no discernible differences in the underlying limb and craniofacial skeletal elements (Fig. 6C–J). In the limb, all skeletal elements were present without evidence of gross morphological alteration. Craniofacially, skeletal preparations and dissection of more than 30 mutant adults revealed no gross changes in the mandible or maxillary bones. There was also no evidence of cleft palate or submucosal cleft palate. In support of this, mutant pup survival was comparable to wild-type and there was no evidence of air in the stomachs of mutant mice – a symptom often associated with suckling difficulties caused by palate or nasal developmental problems. *Tmem26* expression near the base of the developing tongue and in the eyelid led us to examine mice for ankyloglossia, eyes-open-at-birth phenotypes and lens dismorphology but, again, no differences were observed between *Tmem26^Ex2−/Ex2−^* and WT mice. Histological examination of the secondary palate, nasal septum, tongue and eye in 16.5 dpc mice also revealed no differences between *Tmem26^Ex2−/Ex2−^* and WT mice (Fig. 6K,L).

Although bone structure was not obviously altered in *Tmem26^Ex2−/Ex2−^* mice, it was possible that subtle differences in bone shape or size were present but not immediately evident. Other published mouse models with either low or high penetrance of cleft palate often present with alternative craniofacial defects. For example, *Egfr* mutants have a narrow snout and a shortened mandible, whereas *Irf6*, *Dlg*, *Msx1* and *Fgf18* mutants have isolated cleft palate and a shortened snout [14], [15], [16], [17], [18], [19]. Therefore, snout length and width was measured for mutant and WT adults, to identify any subtle disparities (Fig. 6M–O). Comparison of sex-matched littermates revealed no significant difference in snout width or length between mutant and WT mice (n = 18 females or 20 males of each genotype). However, it remains possible that subtle differences in shape or mineralisation of craniofacial bones may be present in *Tmem26^Ex2−/Ex2−^* mice, as has been observed in other models of craniofacial dysmorphology such as *Tbx22* mutant mice [20].

### Concluding comments

Here we describe a novel gene with a complex, regulated pattern of expression that is both spatially and temporally dynamic during mouse development. We also demonstrate that conditional deletion of *Tmem26* in the mouse resulted in no overt embryonic or adult phenotype. While the absence of overt phenotypes suggests that TMEM26 function is not required for embryonic development, it remains possible that more subtle phenotypes may manifest in these mice. Craniofacial development in particular is a complex process, and multiple mechanisms act to buffer the embryo against genetic perturbations and environmental stresses. It is possible that crossing the *Tmem26^Ex2−/Ex2−^* mouse to alternative background strains or genetic mouse models susceptible to facial clefting, or exposure to environmental stresses known to induce such clefts, may reveal a subtle role for TMEM26 in mediating aspects of facial development. In addition, high expression of *Tmem26* in organs associated with immune function suggest a possible role in processes related to the immune response. This possibility is supported by several transcriptional profiling studies [21], [22], [23], [24], but has yet to be investigated functionally.

## Materials and Methods

### Ethics statement

All work involving animals was approved by a University of Queensland Animal Ethics Committee and adhered to strict ethical guidelines (AEC Approval Number IMB/580/08/PHD).

### Whole mount and section in situ hybridisation

Mouse embryos for in situ hybridisation analysis were collected from wild type C57Bl/6 mice. Embryos were fixed with 4% paraformaldehyde in phosphate buffered saline for 4 to 24 hours at 4°C. Embryos to be sectioned were embedded in wax and cut at 8 µm thickness. Whole mount and section in situ hybridisation were performed essentially as previously described [25], [26]. RNA probes were generated by *in vitro* transcription using nucleotides conjugated with either digoxygenin (DIG - Roche Diagnostics, Germany) or ^35^S (Amersham). Three mouse *Tmem26* probes were used for WISH with similar results; whole mount and section in situ hybridisation data presented here are entirely derived from genbank clone BF147423, which localises to the 3′UTR. The other probes were the ORF (1101 bp Refseq NM_177794), and a 3′UTR probe of length 927 bp from base 1081 relative to the start ATG of NM_177794 (Allen Brain Atlas http://www.brain-map.org). Images were captured using either an Olympus BX-51 or an SZX-12 microscope with a DP70 camera and DP Controller software (Olympus Corporation).

### qRT-PCR

qRT-PCR was conducted using SYBR Green reaction mix and a 7900HT (Applied Biosystems) PCR machine with a 384-well format. Most wild type adult tissues and all *Tmem26^Ex2−/Ex2−^* tissues were analysed using several different pairs of qRT-PCR primers (sequence available on request). However, as no obvious variation was observed between the results generated by the different primer pairs, data presented came from one primer pair designed to span exons 2 to 4 (*Tmem26*Fwd;GAAATGCACCATGGAAACC, *Tmem26*Rev; CGGTTCACATACCATGGATAA). qRT-PCR was performed in triplicate on each sample. Primers that amplify *Hprt1* were used as an internal standard (*Hprt1*Fwd; GCAGTACAGCCCCAAAATGG, *Hprt1*Rev; AACAAAGTCTGGCCTGTATCCAA). The mean CT (cycle number at threshold) of the *Hprt1* triplicate of each sample was deducted from the mean CT of the gene of interest triplicate to calculate the ΔCT. Figure 4 shows the mean ΔCT and standard error of the mean (SEM) of biological triplicates for each sample type.

### Targeting construct production and generation of Tmem26^c/c^ and Tmem26^Ex2−/Ex2−^ mice

The *Tmem26* conditional inactivation targeting vector, pN9KO, was based on the backbone vector pOZmod, a derivative of pOzIII. Three genomic regions from the *Tmem26* locus were PCR amplified and ligated into pOZmod, becoming the 5′ homology arm, 3′ homology arm (each being approximately 5 kb) and the 1582 bp targeted region that contains exon2 and exon2a (Targeted region Fwd:GTTTAATTAACCGAGGGTGACTGTGACT, Targeted region Rev: TATGTCGACAAATGATGCCAGAACAAGG, 5′ Arm Fwd; TGGCGCGCCATTGAGGCAGAGGACATC, 5′Arm Rev; ATGGTACCATGATTTCTGCCACAGGGA, 3′ Arm Fwd; GCAGCGGCCGCACTAGATTAAACAACACTTT, 3′ Arm Rev; TGCCCGCGGAAGTGCAAGTCAGTATGTGT). The targeted region was inserted into pOZmod between the 5′ *LoxP* site and the *neomycin* selection cassette (*Neo*). The 3′ *LoxP* site was positioned at the other side of the *Neo* cassette, and therefore introduction of Cre recombinase would mediate the excision of the exon2-2a region and the *Neo* cassette. The 3 *Tmem26* genomic regions were PCR amplified, using the Expand Long Template PCR System (Roche), from DNA extracted from liver tissue of a C57BL/6 mouse.

pN9KO DNA was prepared from *E.coli* by CsCl density gradient as described in [27], and linearised using *Cla*I prior to electroporation. The ES cell line used for pN9KO electroporation (C2) is derived from the C57BL/6 strain and was produced and kindly supplied by Dr Andras Nagy (Samuel Lunenfield Research Institute, Toronto, Canada). Electroporation of ES cells, and selection of cell lines with stably incorporated pN9KO, was performed by standard methods. Neomycin-resistant ES cell lines were subsequently assayed using both PCR (not shown) and Southern blot analyses to identify lines in which pN9KO had undergone correct homologous recombination into the *Tmem26* locus. Southern blots were performed as described [27], using *Eco*RV. The probe used to identify *Tmem26* bands lies outside the genomic regions that contribute to the pN9KO vector. Correctly targeted clones were identified by the presence of two bands, a WT band at 13.6 kb and a targeted band at 10.5 kb.

All injections of ES cells into blastocysts, and implantations of embryos, were performed essentially as described in [25]. Chimeric male offspring were bred to C57BL/6 females, and F^1^ animals were screened using a PCR genotyping protocol. The resultant *Tmem26* conditional inactivation strain (*Tmem26^c^*) was subsequently maintained on a C57BL/6 background. Alternatively, F^1^ *Tmem26^+/c^* male mice were crossed to *CMV-Cre* females (on a C57Bl/6 background) to produce *Tmem26^+/Ex2−^:CMV-Cre* offspring. These offspring were then mated with C57BL/6 animals to remove *CMV-Cre*. The resultant *Tmem26^+/Ex2−^* line was maintained on a C57Bl/6 background. Genotyping for all *Tmem26* alleles used three primers in a triplex reaction (N9GenoS5417; CAGGATTTGCTCTGGCTGC, N9GenoAS5857; ATGTCTGCCCTTTGCCTCC, N9GenoAS9272; TGGAATCCCCATCGCCCTGT) at 60°C annealing temperature. Expected band sizes were *Tmem26^+^*;392 bp, *Tmem26^c^*;440 bp, *Tmem26^Ex2^*;368 bp.

### Skeletal preparations and histology

Adult skeletons were stained with Alcian blue (staining cartilage) and Alizarin red (staining ossified tissues). Adult animals were euthanased with CO_2_, skin was removed and carcasses were placed in boiling water for 30 seconds. Soft tissues were removed manually. Skeletons were fixed in 100% EtOH, 1% glacial acetic acid for 4 days, transferred to acetone for three days, rinsed in distilled H2O and stained for 14 to 28 days in staining solution (1 volume 0.3% alcian blue in 70% EtOH, 1 volume 0.1% alizarin red in 95% EtOH, 1 volume 100% acetic acid, 17 volumes EtOH). Skeletons were then rinsed in distilled water and de-stained in 20% glycerol, 1% potassium hydroxide solution until intensity of stain was appropriate, changing de-staining solution every 2–3 days. After destaining, specimens were transferred through a series of 50%, 80% and 100% glycerol.

Haematoxylin, eosin and toluidine blue histological staining was performed according to standard protocols [28].
